# Facial Paralysis

**Published:** 1862-10

**Authors:** 


FACIAL PARALYSIS.—Dr. Coale reported the case. The patient was a healthy, well-regulated girl, eighteen years of age, who at first noticed that her face was somewhat stiff, and in twenty-four hours completely paralyzed on the left side. There was great distortion on laughing or talking, a staring left eye, and tenderness of the whole left side of the face. The torgue was not at all affected, the disease being confined to the portio dura. No cause could be found for it, unless it were that she had defective teeth in each jaw, as much, however, on one side of the mouth as the other. The treatment consisted of leeches to the place of exit of the nerve, strychnia, etc., but with no benefit. After the lapse of three weeks, she was advised to have her carious teeth removed, and thirteen were accordingly extracted. This was followed by manifest improvement in the course of five days. Electro-galvanism was then gently employed, and the patient recovered.

Dr. Tyler said that several years ago he had under his care a lady with severe sciatica, for which all the usual remedies had been tried in vain. Finding that she had several decayed teeth, he extracted four or five of them, with benefit. The remainder were subsequently drawn, after which the patient had no more pain.

In 1853, a boy of nineteen was brought to the New Hampshire Asylum in a state of mania. Dr. Tyler ascertained that he had had a tooth extracted some time previous, and that one of the fangs had broken off, and remained in the jaw. Suppuration took place, the pus discharging outwardly, and the boy was suddenly attacked with mania. The fang was removed. The fistulous opening closed, and the patient quickly recovered from his mania.

In another case of mania, the patient being a young lady, several decayed teeth were removed. The patient remained to some extent under the influence of the ether, which was given at the operation, for twenty-four hours. After that she was cured of the mania.

These facts have led Dr. Tyler to regard decayed teeth as of great importance in connection with nervous disorders.—*Extracts from the Records of the Boston Society for Medical Improvement, in Boston Med. & Surg. Jour.*

